# Impact of Application of Multifunction Electrode (MFE) Pads on Cardiopulmonary Resuscitation Quality

**DOI:** 10.1155/2020/2675214

**Published:** 2020-10-19

**Authors:** Radosław Zalewski, Mateusz Puślecki, Łukasz Szarpak, Tomasz Kłosiewicz, Marek Dąbrowski, Bartłomiej Perek

**Affiliations:** ^1^Department of Medical Rescue, Chair of Emergency Medicine, Poznan University of Medical Sciences, Collegium Adama Wrzoska Rokietnicka Street 7, Poznan 60-806, Poland; ^2^Department of Cardiac Surgery and Transplantology, Chair of Cardiac and Thoracic Surgery, Poznan University of Medical Sciences, Dluga Street 1/2, Poznan 61–848, Poland; ^3^Maria Sklodowska-Curie Medical Academy in Warsaw, 12 Solidarnosci Av., 03-411 Warsaw, Poland; ^4^Chair and Department of Medical Education, Poznan University of Medical Sciences, Collegium Adama Wrzoska Rokietnicka Street 7, Poznan 60-806, Poland

## Abstract

**Background:**

Early defibrillation and high-quality chest compressions are crucial in treatment of sudden cardiac arrest (SCA) subjects. The aim of this study was to assess an impact of defibrillation methods on cardiopulmonary resuscitation (CPR) quality.

**Methods:**

A randomized simulation cross-study was designed, in which 100 two-person paramedical teams participated. Two 10-minute scenarios of SCA in the mechanism of ventricular fibrillation were analysed. In the first one, teams had at their disposal defibrillator with hard paddles (group C), whereas in the second one, adhesive electrodes were used (group MFE). The CPR quality was evaluated on the basis of the chest compression parameters (rate, depth, recoil, compression fraction (CCF), and no-flow time), airways patency achievement, and successful emergency drug administration.

**Results:**

Substituting standard hard paddles with adhesive electrodes led to an increase in CCF (77% vs 73%; *p* < 0.05), higher rate of complete chest recoil, and a decrease in no-flow time (6.0 ± 1.1 vs. 7.3 ± 1.1; *p* < 0.001). The airway patency was ensured sooner in group MFE (271 ± 118 s vs. 322 ± 106 s in group C; *p* < 0.001). All teams in scenario with adhesive electrodes were able to administer two doses of adrenaline, meanwhile only 74% of them in group C (*p* < 0.001). Moreover, in 8% of group C scenarios, paramedics did not have enough time to administer amiodarone.

**Conclusion:**

Our simulation-based analysis revealed that use of adhesive electrodes during defibrillation instead of standard hard paddles may improve the quality of CPR performed by two-person emergency team.

## 1. Introduction

Sudden cardiac arrest (SCA) in prehospital settings affects an increasing number of patients. Only in the United States of America, 420,000 OHCA cases are reported annually [1]. The latest data collected in 27 European countries by in EuReCa ONE registry showed the prevalence of out-of-hospital cardiac arrest (OHCA) ranged from 19.0 to 104.0 cases per 100,000 [2].

All scientific societies of medical specialists in emergency medicine agree that SCA requires active and proper interventions to restore circulation [3, 4]. It is a general consensus that if defibrillation procedure is implemented faster, spontaneous circulation is more likely to return. The guidelines of the European Resuscitation Council emphasize that every minute of defibrillation delay reduces the chance of OHCA survival by 10–12%. However, the rapid implementation of defibrillation can increase survival by up to 50–70% [3, 4]. Public defibrillation programs are being developed in many countries. In Japan, survival of OHCA with favourable neurological results has increased significantly due to AED accessible in public places [5].

Another aspect of resuscitation is a permanent shortage of public funds to cover all requirements of national healthcare system resulted, among many others, and in reduction in number of paramedics in the emergency medical teams. In many developed countries, a standard consists of a two-person team well-equipped with sophisticated and novel devices, including defibrillators compliant with adhesive electrodes.

The aim of our analysis was to assess the impact of two types of defibrillation methods, namely, standard hard paddles and adhesive electrodes, on the quality of cardiopulmonary resuscitation.

## 2. Materials and Methods

### 2.1. Study Design and Participants

A randomized simulation cross-study was designed, in which 100 two-person teams of paramedics participated. They were randomly selected from a group of 300 professionally active paramedics with a minimum of five-year experience. The age of the study participants ranged from 27 to 55 years. All paramedics took part in the study voluntarily. The study protocol was approved by the local bioethical committee (No. KB 1075/19). The research project did not have any source of funding. The randomization procedure was prepared with a use of a free online randomization tool (http://www.randomizer.org). The allocation to the first group was randomized.

### 2.2. Study Protocol

Each team participated in two 10-minute simulated scenarios of SCA with the persistent ventricular fibrillation rhythm. In the first one, teams used defibrillator with hard paddles (group C), whereas in the second one, paramedics had only adhesive electrodes at their disposal (group MFE). To standardize the operation of all teams, they were forced to use the same method of defibrillation—a model that involves charging the defibrillator just before performing the analysis. Paramedics participating in the study received information that all activities were to be carried out in real time. The teams had 15-minute breaks between the scenarios. All simulations were supervised by qualified medical simulation instructors.

### 2.3. Simulator

In our study, high-fidelity simulator (Resusci Anne QCPR, Laerdal Medical, Stavanger, Norway) was used. This dummy registered chest compression parameters. Moreover, effects of the other medical emergency procedures such as intubation, mechanical ventilation, and defibrillation were visible on this dummy. Its hands were filled with blood, so paramedics could see whether intravenous angiocatheters were inserted correctly.

### 2.4. Equipment and Medicines

Before starting the scenario, each team was thoroughly acquainted with the available equipment and work environment. A standard defibrillator (M-Series, ZOLL, Chelmsford, USA) was equipped with manual paddles or adhesive electrodes. The electrodes for each use were originally packed. Before commencing the scenario, the device was thoroughly discussed, and team members could practice on it. Paramedics were equipped with a medical bag that contained necessary medications, liquids, needles, catheters, syringes, devices for clearing the respiratory tract, patches, and boxes for sharp objects. This equipment could be arranged individually in a bag by each team.

### 2.5. Assessed Parameters of CPR

Available parameters of chest compressions quality were recorded by means of the Laerdal PC Skill Reporting System program version 2.0 (Laerdal Medical, Stavanger, Norway). The following parameters such as CCF, average no-flow time, correct hand placement, total number of compressions, average depth of compressions, percentage of appropriately deep compressions, percentage of compressions with adequate chest relaxation (chest recoil), the average compressions rate, and the percentage of the correct compressions rate were recorded. Additionally, the duration of defibrillation itself was measured in both groups.

Moreover, we evaluated time to assure airways patency (i.e., successful introduction of supraglottic airways device (SAD)) and to administer the consecutive doses of drugs commonly injected during CPR such as amiodarone and adrenaline.

### 2.6. Data Management and Statistical Analysis

The data recorded by the reporting PCI system incorporated in simulator were entered into a previously prepared Excel spreadsheet. Regarding continuous data, they were checked for normality with the use of the Shapiro–Wilk W test. Those satisfying the criteria of normal distribution were expressed as the mean with standard deviation (sd), and paired Student's *t*-test was applied to estimate differences between groups. Continuous data that cannot be assumed to be normally distributed were presented as the medians (25th and 75th percentiles) and then analysed by means of the Wilcoxon matched-pairs test. The categorical variables were expressed as the numbers (*n*) with percentage (%), and then, they were compared between subgroups with the use of the Yates corrected *χ*^2^ test. A *p* value below 0.05 was considered of statistical significance. The statistical analysis was performed with the use of Statistica 10.0 for Windows (StatSoft, Inc., Tulsa, OK, USA).

## 3. Results

Replacing standard defibrillation paddles (group C) with MFE Pads led to an improvement in chest compression quality estimated on the base of CCF, no-flow time, and parameters of compressions themselves such as optimal rate, depth, and recoil after every compression. CCF was significantly higher in group MFE, while no-flow time was lower compared to group C. Detailed results of statistical analysis regarding chest compression adherence to the recommendations are outlined in Table 1.

### 3.1. Defibrillation

In all cases of group MFE, defibrillation was performed five times within 10-minute scenarios, whereas 86% in group C cases (*p*=0.001). Moreover, all except one application was faster in group MFE than in C. The detailed data are presented in Table 2.

### 3.2. Different Methods of Defibrillation and Airway Patency

In the group MFE, SAD insertion manoeuvres were initiated significantly (*p* < 0.05) earlier after CPR initiation (213 ± 112 s) and lasted shorter (57 ± 27 s) than in group C (243 ± 100 s and 79 ± 39 s, respectively). Consequently, airway patency was assured faster in the earlier group (271 ± 118 s vs. 322 ± 106 s) (*p* < 0.001).

### 3.3. Impact of MFE Pads on Drug Administration

**Figure 1:**
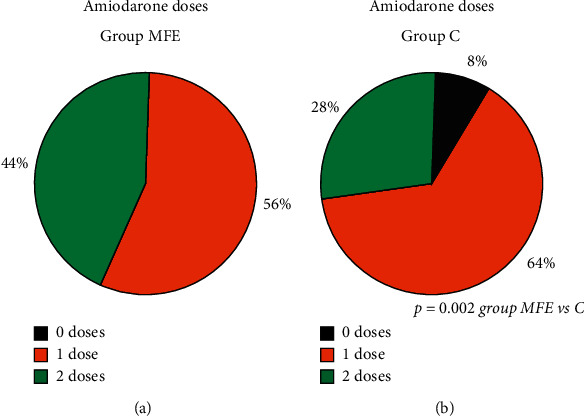
Number of amiodarone injections in both groups.

**Figure 2:**
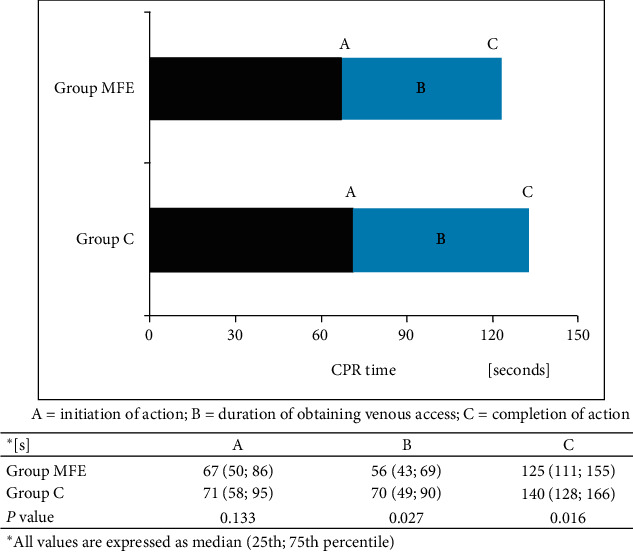
Obtaining of peripheral venous access in the studied groups.

Paramedics injected two doses of adrenaline in all scenarios with MFE pads, whereas, in group C, 74% (*p* < 0.001). Similarly, significant differences were noted regarding administration of amiodarone. Of note, in 8% of scenarios in group C, no single doses of amiodarone, although prepared, were used (Figure 1). It could have resulted from later obtaining venous access in the latter group (Figure 2). It should be stressed that the action of peripheral venous access insertion was started only slightly later but lasted longer in group C than group MFE.

## 4. Discussion

It is commonly accepted and supported by valid guidelines that high-quality chest compressions and early defibrillation have the greatest impact on SCA survival. Both actions should be implemented as soon as possible [6]. Their initiation and optimal continuation may be a real challenge if the must be performed by only two-person emergency teams. Thus, application of some devices reducing involvement of both paramedics may be very helpful. In this study, importance of adhesive electrodes compliant with standard defibrillator on CPR quality has been tested. Having in mind that CPR confines many single actions performed by paramedics, many of them such as chest compressions, defibrillation, airways patency, access to peripheral vein, and emergency drugs administration were included in our analysis.

Strictly defined parameters of high quality of chest compressions are associated with optimal frequency of return of spontaneous circulation (ROSC) and survival rate. The frequency should be kept between 100 and 120 compressions per minute while the depth 5 through 6 cm. The reverse association between rate and depth was noted before [7, 8]. Moreover, the experimental studies in animal models revealed the importance of chest wall recoil. This causes negative chest pressure formation which facilitates venous return to the heart. By generating the phenomenon described above, it is possible to increase coronary perfusion pressure [9]. It was found in our study that although chest compressions performed by experienced paramedics in simulated scenarios were far from optimal, application of self-adhesive electrodes in the MFE group was linked to marked improvement in correct compression depth and chest recoil. The guidelines also emphasize a significant value of minimizing breaks during chest compressions. The American Heart Association experts have established a parameter called CCF (chest compression fraction) defined as the percentage of time spent on uninterrupted chest compressions. The optimal CCF value should be at least 80% [10]. This value is also confirmed by clinical experience, which shows that the best prehospital cardiac arrest survival results are achieved while maintaining CCF values within 81–100% [11]. Our study showed an increase in the CCF and corresponding decrease in no-flow time in group MFE. It must be stressed that, even in the latter subset, average value of 77% was too low to ensure optimal chest compression quality. A significant improvement regarding these aforementioned parameters may result from no need of placing standard ECG electrodes on a chest to proceed with analysis and consecutive defibrillation.

Previous studies showed that the highest survival rate was recorded among patients with diagnosed VF/pVT when chest compressions were initiated and defibrillation was carried out within the first 3–5 minutes following the loss of consciousness [12]. In case of unavailability of defibrillator at the site of OHCA, mechanical resuscitation before professional medical intervention increases survival rate of SCA subjects. Our study showed that all defibrillations were performed earlier in group MFE. Exception was the fifth defibrillation, but it must be stated that, in group C, it was performed in 86% of scenarios while, in 100%, in group MFE.

Up to now, three main models of defibrillator charging and chest compression have been developed [13]. In the first one, chest compressions are stopped to analyse heart rhythm, charge the defibrillator, and eventually defibrillation. After the latter, one compression is resumed. The pauses in chest compression are the longest. In the second one, compressions are suspended just for heart rhythm analysis and defibrillation itself. During charging of defibrillation, taking place after rhythm analysis, the chest is compressed. In the last model, charging of defibrillator is done blindly even before rhythm analysis. It was shown that irrespective of the described above model, the breaks in chest compressions depended on a form of defibrillation devices. If paddles are employed, key compression and defibrillation times will depend on place of charging, i.e., on the device or on the patient's chest. If self-adhesive defibrillation pads are used for defibrillation, charging can be performed blindly before or after the rhythm analysis [13, 14]. Additionally, their application can significantly reduce the delay in performing the defibrillation [14]. Our analysis confirmed this finding. This is probably the main mechanism underlying the clinical observation that self-adhesive pads during cardiopulmonary resuscitation increase the frequency of ROSC and in-hospital survival. More advantages of these electrodes have been advocated. They are invaluable for carrying out cardioversion or percutaneous stimulation in cardiac surgical patients during and soon after invasive open-chest procedures [15].

Patients with OHCA whose proper heart rate was restored require to be transported to hospital as soon as possible. They are at high risk of cardiac arrest or other cardiac arrhythmias. Application in them of self-adhesive pads for transport is extremely important as defibrillation can be delivered immediately, or transdermal stimulation can be initiated without any delay [16] The use of self-adhesive pads can significantly facilitate other necessary procedures carried out by paramedics during CPR, particularly in challenging prehospital conditions characterized by the pressure of a large number of distractions such as bystanders present near the patient, noise at the place of incident, fatigue, poor nutrition, or lack of sleep. The human factor in this work is extremely important, and simplification of procedures allows paramedics to focus on the priorities of action [17]. We found in our study that self-adhesive electrodes had also positive impact of other procedures. Paramedics in MFE scenario decided faster about SAD application, and airways patency was achieved faster in this subset. The access to peripheral vein went smoother in group MFE; the majority of study participants were able to complete the algorithm of emergency drugs administration, whereas it was not possible in many scenarios in the control group. Teams using standard paddles did not have enough time to inject the second dose of amiodarone.

The results of the aforementioned studies were reflected in the current guidelines, both AHA and ERC that recommended the use of self-adhesive pads. This is argued by the numerous benefits of defibrillation using paddles [18, 19]. On the other side, the self-adhesive pads have also potential disadvantages. If they are attached to the anterior aspect of the chest wall, they can interfere with radiographic visualization of the heart. To overcome this problem, the pads of different shapes have been designed [20]. Monitoring of heart rate with the use of them, which was previously used for defibrillation, showed that, during the discharge, the electrolyte gel gets polarized. This process creates a risk that a false asystole may appear on the ECG monitor. The authors of this study recommend the use of monitoring cable to confirm the actual heart rhythm [21].

At last, costs of their routine use are a huge financial problem. Pinkham-Reidy et al. on the basis of cost-effectiveness analysis showed that routine use of these pads resulted in high costs for the healthcare system and should be applied only if indicated [22].

## 5. Conclusion

Our simulation-based analysis revealed that use of adhesive electrodes during defibrillation instead of standard hard paddles may improve the quality of cardiopulmonary resuscitation performed by two-person emergency team.

## Figures and Tables

**Table 1:** Selected parameters of chest compression in both groups.

CC parameters^*∗*^	Group MFE (*n* = 100)	Group C (*n* = 100)	*p* value
CC rate (minute)	121 ± 9	122 ± 8	0.691
Correct CC rate (%)	36 (16; 59)	33 (12; 47)	0.064
CC depth (mm)	55 ± 5	52 ± 4	<0.001^#^
Correct CC depth (%)	53 (45; 60)	45 (32; 55)	<0.001^#^
Correct chest recoil (%)	32 (22; 48)	19 (9; 38)	0.001^#^
Correct hands position (%)	100 (99; 100)	100 (98; 100)	0.372
CCF (%)	77.0 ± 6.5	73.1 ± 7.1	<0.001^#^
No-flow time (sec)	6.0 ± 1.1	7.3 ± 1.1	<0.001^#^

^*∗*^Continuous variables are expressed as means with standard deviations (with normal distribution) or medians (25th; 75th percentile). ^#^Statistically significant differences between studied groups (*p* < 0.05). C = control; CC = chest compression; CCF = chest compression fraction; MFE = multifunction electrode.

**Table 2:** Time of consecutive applications of defibrillations.

No.^*∗*^	Group MFE	Group C	*p* value
1^st^ defibrillation	39.0 ± 10.6 (100)^*∗∗*^	50.4 ± 16.8 (100)	<0.001
2^nd^ defibrillation	166.6 ± 23.0 (100)	179.8 ± 28.1 (100)	0.011
3^rd^ defibrillation	290.2 ± 24.0 (100)	313.1 ± 39.6 (100)	0.001
4^th^ defibrillation	413.3 ± 25.1 (100)	440.9 ± 49.1 (100)	0.001
5^th^ defibrillation	535.9 ± 24.5 (100)	535.1 ± 63.0 (86)	0.785

^*∗∗*^Data are expressed as mean with standard deviation (sd); ^*∗*^number of analysed defibrillations. C = control; MFE = multifunction electrode.

## Data Availability

The data used to support the findings of this study are available from the corresponding author upon request.
